# Usefulness of Non-Skin Samples in the PCR Diagnosis of Mpox (Monkeypox)

**DOI:** 10.3390/v15051107

**Published:** 2023-04-30

**Authors:** Sonia Algarate, Jessica Bueno, María J. Crusells, Mariano Ara, Henar Alonso, Elena Alvarado, María Ducons, Sara Arnal, Rafael Benito

**Affiliations:** 1 Hospital Clínico Universitario Lozano Blesa de Zaragoza, Microbiology Department, University of Zaragoza, Instituto de Investigación Sanitaria de Aragón, 50009 Zaragoza, Spain; scajo@unizar.es; 2 Hospital Clínico Universitario Lozano Blesa de Zaragoza, 50009 Zaragoza, Spain; 3 Hospital Clínico Universitario Lozano Blesa de Zaragoza, Microbiology Department, University of Zaragoza, 50009 Zaragoza, Spain; 4 Microbiology Department, University of Zaragoza, 50009 Zaragoza, Spain

**Keywords:** monkeypox, mpox, PCR, diagnosis, serum

## Abstract

Cases of mpox have been reported in several European countries, including Spain. Our objective was to evaluate the usefulness of serum and nasopharyngeal samples for diagnosis of mpox. The presence of MPXV DNA was studied using real-time PCR (Cer*Test* Biotec, Zaragoza, Spain) in 106 samples from 50 patients: 32 skin, 31 anogenital, 25 sera, and 18 nasopharyngeal/pharyngeal, in the Hospital Clínico Universitario of Zaragoza (Spain). Sixty-three samples from twenty-seven patients were MPXV PCR-positive. The real-time PCR Ct values in the anogenital and skin samples were lower than serum and nasopharyngeal samples. More than 90% of anogenital (95.7%), serum (94.4%), and skin (92.9%) samples were real-time PCR-positive. Eighteen (66.7%) of the twenty-seven patients who were MPXV PCR-positive had antecedents or presented with one to three sexually transmitted infection (STI) agents. Our results indicate that the use of serum samples can help facilitate the diagnosis of MPXV infections.

## 1. Introduction

In 2022, cases of human monkeypox, now named mpox following WHO recommendations [1], have been reported in several European countries, including Spain [2]. By 27 December 2022, in Spain, 7404 cases were detected, the majority of which were linked to sexual intercourse, and 74 of which were identified in Aragón [3]. This unknown disease led to a consequent state of alarm.

Monkeypox virus (MPXV) is an enveloped double-stranded DNA virus belonging to the *Orthopoxvirus* genus of the *Poxviridae* family. Two phylogenetically distinct clades of MPXV have been identified: the Central African (Congo Basin) clade and the West African clades [4], now proposed as clades I and II, respectively [5]. Typically, the Central African MPXV is associated with more severe disease, higher mortality, and more frequent human-to-human transmission.

Mpox is an endemic disease in some areas of central and western Africa, where it circulates among wildlife, but can occasionally be transmitted to humans. Its reservoir in endemic areas is still unknown, but it is suspected that rodents may be involved in its transmission [6].

MPXV is transmitted to humans through contact with live or dead infected animals, by bite or scratch, bush meat preparation, and direct contact with body fluids, lesions, or contaminated material [7,8]. Human-to-human transmission of MPXV occurs mostly through large respiratory droplets during direct and prolonged face-to-face contact, but also by direct contact with body fluids (including in sexual intercourse, as is the case with the MPXV responsible for the 2022 outbreak) or with contaminated objects, such as bedding or clothing. After viral entry from the oropharynx, nasopharynx, or intradermal route, MPVX replicates locally, spreads to local lymph nodes, and from there causes viremia, arriving in other organs [9]. This occurs during the incubation period, which can range from 5 to 21 days, but can be shorter [7,8]. Symptoms appear during a second viremia after 1 to 2 days of prodromal symptoms, such as fever and lymphadenopathy, but they may be absent.

Human mpox often begins with a combination of the following symptoms: fever, headache, chills, asthenia, adenopathies, and back and muscle pain. One to three days after the onset of fever, the patient develops a cutaneous rash, which often first presents as macules, evolving successively to papules, vesicles, pustules, and scabs. Lesions are often single in the current outbreak. Mpox can also manifest as oropharyngeal lesions or as proctitis. The main difference between smallpox and monkeypox viruses is that the latter cause lymphadenopathy (e.g., in the cervical or inguinal region), while the former and the chickenpox virus usually do not. For most people, mpox is a self-limited disease, typically lasting two to four weeks and resulting in complete recovery [10].

In the current context, rapid identification of cases, clusters, and the sources of infection are very important for the optimal management of the problem. In this sense, polymerase chain reaction (PCR) is the preferred laboratory test given its rapidity, accuracy, and sensitivity [4,11,12,13,14].

Our objective was to evaluate the usefulness of serum and nasopharyngeal samples in the molecular diagnosis of mpox.

## 2. Materials and Methods

A total of 106 clinical samples from 50 patients, 38 male and 12 female, aged between 11 to 62 years (mean = 34.4 years), with skin lesions suspected of MPXV infection or by contact study were analyzed in the Hospital Clínico Universitario of Zaragoza (Spain). Samples were obtained between May and September 2022. Non-serum samples were obtained with a swab and placed in a viral transport and preservation medium with virus inactivator (Biocomma, ShenZheng, China). The presence of MPXV DNA was studied using real-time PCR. Out of 50 patients, 32 were Spanish and 18 were immigrants. The analyzed samples were: 32 skin, 31 anogenital, 25 sera, 17 nasopharyngeal, and 1 pharyngeal. Samples were obtained between 1 and 19 days after the onset of symptoms (mean = 5 days ± 4.2). All samples were collected by specialized staff and their analysis was carried out in the first 24–48 h. Until analysis, samples were properly stored at 4 °C.

To perform the real-time PCR, the VIASURE *Monkeypox Virus* Real Time PCR Detection Kit (Cer*Test* Biotec, S.L., San Mateo de Gállego, Zaragoza, Spain) and the VIASURE 48 Real Time PCR System (Cer*Test* Biotec, S.L.) were used. Nucleic acid extraction was performed with the automated nucleic acid extraction system MagLEAD12gC using the MagDEA^®^Dx SV reagents (Precision System Science Co., Ltd., Matsudo, Japan). The detection of MPXV DNA was performed by amplifying a conserved region of *G2R* and *F3L* genes, both in the same channel, using specific primers and fluorescent-labelled probes. This molecular assay detects both clades of MPXV. The manufacturer’s instructions were always followed, which considers Ct ≤ 40 to be MPXV DNA-positive. Results were obtained in 120 min approximately. All steps requiring specimen manipulation were conducted within a certified biosafety cabinet. The VIASURE kits were kindly provided by Cer*Test* Biotec, S.L.

The protocol was approved by the Clinical Investigation Ethics Committee of Aragón (PI22/409) and was performed in accordance with the ethical standards noted in the 1964 Declaration of Helsinki.

Statistical analysis: Figures are given in absolute numbers and percentages. Quantitative and qualitative variables are described as mean with SDs, proportions, or range. Bivariate comparisons of quantitative and qualitative variables were performed using the two tailed Mann–Whitney U test and χ^2^-Fisher test. Statistical significance was set at *p* < 0.05 for all calculations. Data analysis was performed using SPSS v.19 (IBM, Chicago, IL, USA).

## 3. Results

**Figure 1:**
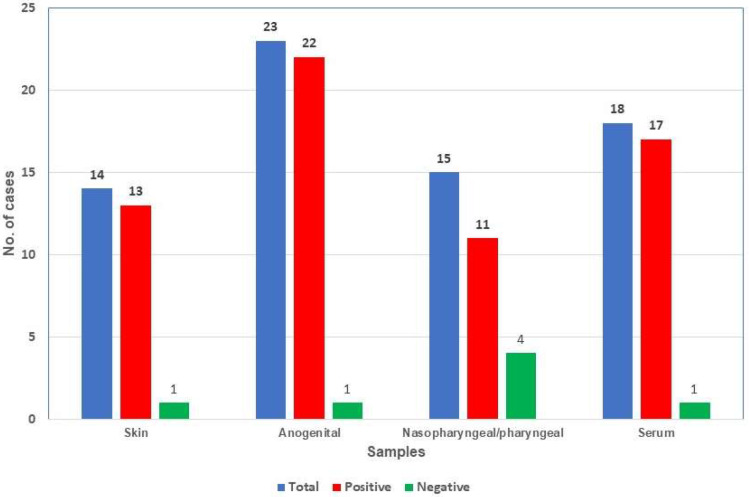
Results of MPXV infected patients.

Table 1 and Figure 1 show the real-time PCR results. Table 1 also expresses the mean of PCR Ct values. Sixty-three samples from twenty-seven patients were MPXV PCR-positive. These 27 patients, 25 men and 2 women, were classified as infected by MPXV because they were PCR-positive in one or more samples. These samples were obtained between 1 to 10 days after the onset of symptoms (mean 4.5 days). Skin, anogenital, and serum samples were the more efficient for mpox diagnosis. Nasopharyngeal/pharyngeal samples provided more negative results.

The real-time PCR Ct values in the anogenital and skin samples were lower than serum and nasopharyngeal samples, with statistically significant differences (which suggests higher viral load) (Table 1). Nevertheless, these data were influenced by the date the sample was obtained.

Serum samples of 18 out of the 27 infected patients were obtained, showing only 1 (3.7%) to be a negative PCR result. This patient (case 9) was a male, 24 years old with genital lesions, and PCR-positive in nasopharyngeal swab and genital samples (Table 2). His serum sample was collected two days after the onset of symptoms.

One of the positive patients was a 49-year-old Ecuadorian woman with lesions on the face, limbs, chest, and vulva, lymphadenopathy, and positive real-time PCR in vulvar lesions and in serum, with Ct of 21.3 and 30.9, respectively, at 3 days after the onset of symptoms. In this case, PCR was not carried out on the skin lesions. The second positive female was a 36-year-old Spanish woman with lesions on the face, limbs, and chest, lymphadenopathy, and pharyngitis, and positive real-time PCR on the skin, in the nasopharyngeal swab, and in serum, with a Ct of 21.8, 18.9, and 31.5, respectively, at 7 days after the onset of symptoms.

Eighteen (66.7%) of the twenty-seven patients who were MPXV PCR-positive had antecedents or presented with one to three sexually transmitted infection (STI) agents: *T. pallidum* (*n* = 8), *N. gonorrhoeae* (*n* = 5), *M. genitalium* (*n* = 4), *C. trachomatis* (*n* = 2), HIV (*n* = 8), HPV (*n* = 1), HVA (*n* = 1), or HBV (*n* = 1).

Thirty-six samples of twenty-three patients were MPXV PCR-negative (Table 3). These 23 patients were classified as non-infected by MPXV because they were PCR-negative in one or more samples from the same or different location. These samples were obtained between 1 to 19 days after the onset of symptoms (mean 6.0 days).

Negative samples from 21 cases were sent to the Instituto de Salud Carlos III, Madrid, Spain (samples 1 and 2), or to Hospital Universitario Miguel Servet of Zaragoza, Spain (19 samples), who confirmed the results.

Thirteen (56,5%) of the twenty-three patients with negative MPVX PCR presented or had a history of one to four STIs due to these agents: *T. pallidum* (*n* = 5), *N. gonorrohoeae* (*n* = 3), *M. genitalium* (*n* = 1), *C trachomatis* (*n* = 2), HIV (*n* = 4), HPV (*n* = 2), HSV-1 (*n* = 3), HSV-2 (*n* = 1), HBV (*n* = 1), or HCV (*n* = 1). Another patient was positive for chickenpox.

In this study, there were no significant differences between the STI frequencies in the positive and negative MPXV populations.

In 21 of the 27 MPVX PCR positive patients, between 2 and 4 samples of different locations were obtained on the same day. In 15 (71.4%) of the 21 cases, there was positivity in all of them, but 6 (28.6%) presented a PCR-negative result in some of the samples: 1 skin, 1 serum, 4 nasopharyngeal, and 1 anal (Table 2).

Case 3 was PCR-positive in the nasopharyngeal sample but not in the pharyngeal sample, both obtained three days after the onset of symptoms. Case 9 was PCR-positive in two genital samples and one nasopharyngeal sample, but not in serum, all of which were obtained two days after the onset of symptoms. Case 22 was PCR-positive in skin and in serum, but not in nasopharyngeal samples, all of which were obtained four days after the onset of symptoms. Case 23 was positive in skin and in serum, obtained nine days after the onset of symptoms, but not in the nasopharyngeal sample obtained ten days after the onset of symptoms. Case 30 was positive in two genital samples but not in serum nor anal locations, all of which were obtained three days after the onset of symptoms. Case 47 was positive in genital, nasopharyngeal, and serum samples, but not in skin; all of which were obtained seven days after the onset of symptoms.

## 4. Discussion

Human mpox lesions, especially in their generalized form or in immunocompromised individuals, can be misdiagnosed because they may be confused with chickenpox, herpes simplex infection [11], or smallpox [15,16]. Therefore, specific real-time PCR diagnosis is a very useful tool to diagnose mpox.

We used a real-time PCR to diagnose 50 cases with suspicion of mpox. Several samples were obtained from the same patient: skin, anal, genital, nasopharyngeal, pharyngeal, or serum. Out of 50 cases, 27 were confirmed and 23 were discarded.

Real-time PCR was positive in the samples from all the locations studied in 71.4% of the infected patients. In some cases, there were discrepancies in the results from the different samples collected from the same patient, a situation also described by other authors [17] and which is related to a different viral load. The differences in the Ct are related to the date the sample was obtained.

Negative results could be due to the absence of MPXV infection, the sample being taken at the wrong time, the sample being taken incorrectly [18], or viral intermittent shedding [17]. In these patients, 4 HSV infections, 2 HPV infections, and 1 VZV infection were detected. Our samples were taken in a time range compatible with the virus elimination period (2 to 16 days) previously described [17]. The existing alarm caused a high degree of suspicion in the at-risk population, perhaps due to co-infection with other infectious agents, or with a rash disease of unknown origin, which probably led to the taking and sending of unnecessary samples for MPXV to the laboratory. Most of the negative samples were sent to national or community reference centers for confirmation.

Respiratory samples (pharyngeal and nasopharyngeal) were the ones that showed the highest number of false negative results, probably due to the greater difficulty in their standardization and for the reasons previously mentioned.

There was only one skin sample and one serum sample with a negative result. These samples were taken early or late, which would agree with the evolution of the lesions described in the bibliography [13]. According to these authors, the median of number of days from onset of fever to onset of rash is about 2 days. Later, the rash progresses slowly over a 2-to-3-week period, appearing as vesicles, pustules, and crusts. Negative cases emphasize the need to resort to microbiological diagnosis for a correct classification of patients.

The studied population included mostly men who have sex with men (MSM). In this type of population, STIs are common, so they must be ruled out in the diagnostic process. The high frequency of other STIs in the studied patients, and with frequencies without significant differences, 66.7% in MPXV-positives and 56.5% in MPVX-negatives, confirms that the patients studied are a population at-risk for STIs, in whom MPXV investigation was indicated.

Two women were MPXV DNA-positive, although the most diagnosed population was men, potentially due to homosexual intercourse. This shows that the suspicion of infection should also be directed at the female population.

Real-time PCR is a very good diagnostic tool for the easy and rapid detection of MPXV infection, which is vital for patient management and for implementing public health measures [18].

Several types of samples can be used for MPXV molecular diagnosis [17]. Previous papers have both reported the detection of the virus in the blood of some patients [19,20,21], but our study includes a larger number of serum samples. Our work confirms these previous results and supports the usefulness of serum in the diagnosis of mpox.

Serum sampling can be very useful in the diagnosis of MPXV infection because it is easy to obtain, its content is uniform, it is not affected by the way or the area of the lesion it was taken, it is applicable to contacts without lesions, to patients with old lesions and patients in whom it is difficult to obtain a skin sample, it allows ruling out other STI agents serologically (HBV, HCV, HIV, HAV, EBV, *Treponema pallidum*...), and in our study it was positive in most of the cases of mpox in which we had such a sample.

## 5. Conclusions

Our results indicate that the use of serum samples can help facilitate the diagnosis of MPXV infections. In addition, a serum sample can be used to rule out other STIs by serological diagnosis.

## Figures and Tables

**Table 1:** Results of MPXV PCR of infected patients.

Samples	No.	Days after the Onset of Symptoms (Mean)	Positive (%)	Mean Ct
Skin	14	2–9 (4.9)	13 (92.9)	26.0
Anogenital	23	1–7 (3.7)	22 (95.7)	23.7
Nasopharyngeal/Pharyngeal	15	2–10 (4.8)	11 (78.6)	31.3
Serum	18	1–9 (5.1)	17 (94.4)	34.6
Total	70	1–10 (4.5)	63 (90.0)	28.4

**Table 2:** Results in infected patients with several samples.

Case	Skin 1	Skin 2	Genital 1	Genital 2	Anal	NP	Pharyngeal	Serum	Days after the Onset of Symptoms
3						POS	NEG	POS	3
4	POS							POS	4
5			POS	POS				POS	6
7	POS	POS				POS		POS	3
8			POS					POS	6
9			POS	POS		POS		NEG	2
10	POS		POS			POS			2
13	POS					POS		POS	6
14			POS					POS *	3 and 5 *
21			POS					POS	7
22	POS					NEG		POS	4
23	POS					NEG **		POS	9 and 10 **
24	POS					POS		POS	4
26	POS		POS			POS		POS	4
29			POS			POS		POS	7
30			POS	POS	NEG	NEG			3
31			POS	POS				POS	1
36			POS	POS				POS	3
43	POS		POS ***			POS			4 and 5 ***
46	POS					POS		POS	7
47	NEG		POS			POS		POS	7

NP = Nasopharyngeal. Asterisks refer to the day the respective sample was taken.

**Table 3:** Data of MPXV PCR in non-infected patients.

Samples	No.	Negative (%)	Days after the Onset of Symptoms (Mean)
Skin	18	18 (100)	1–19 (5.4)
Anogenital	8	8 (100)	2–14 (9.6)
Nasopharyngeal	3	3 (100)	1–4 (2.0)
Serum	7	7 (100)	1–14 (8.6)
Total	36	36 (100)	1–19 (6.0)

## Data Availability

All data are contained within the article; for further information please contact the corresponding author.
